# The 2017 Women’s Health Initiative study and use of hormone therapy: an emulated repeated cross-sectional study

**DOI:** 10.1186/s12889-024-19089-2

**Published:** 2024-06-24

**Authors:** Chen-Han Chueh, Pei-Kuan Ho, Wai-Hou Li, Ming-Neng Shiu, I.-Ting Wang, Yu-Wen Wen, Yi-Wen Tsai

**Affiliations:** 1 Institute of Health and Welfare Policy, National Yang Ming Chiao Tung University, Taipei, Taiwan; 2 Department of Obstetrics and Gynecology, Chen Hsin General Hospital, Taipei, Taiwan; 3 Department of Pharmacy, National Yang Ming Chiao Tung University, Taipei, Taiwan; 4 Department of Biomedical Sciences, Chang Gung University, Taoyuan, Taiwan

**Keywords:** Women’s Health Initiative, Hormone therapy, Menopause, Repeated cross-sectional, Simple random sampling, Interval-censored data

## Abstract

**Background:**

Hormone therapy (HT) use among menopausal women declined after negative information from the 2002 Women’s Health Initiative (WHI) HT study. The 2017 post-intervention follow-up WHI study revealed that HT did not increase long-term mortality. However, studies on the effects of the updated WHI findings are lacking. Thus, we assessed the impact of the 2017 WHI findings on HT use in Taiwan.

**Methods:**

We identified 1,869,050 women aged 50–60 years, between June and December 2017, from health insurance claims data to compare HT use in the 3 months preceding and following September 2017. To address the limitations associated with interval-censored data, we employed an emulated repeated cross-sectional design. Using logistic regression analysis, we evaluated the impact of the 2017 WHI study on menopausal symptom-related outpatient visits and HT use. In a scenario analysis, we examined the impact of the 2002 trial on HT use to validate our study design.

**Results:**

Study participants’ baseline characteristics before and after the 2017 WHI study were not significantly different. Logistic regressions demonstrated that the 2017 study had no significant effect on outpatient visits for menopause-related symptoms or HT use among women with outpatient visits. The scenario analysis confirmed the negative impact of the 2002 WHI trial on HT use.

**Conclusions:**

The 2017 WHI study did not demonstrate any impact on either menopause-related outpatient visits or HT use among middle-aged women in Taiwan. Our emulated cross-sectional study design may be employed in similar population-based policy intervention studies using interval-censored data.

**Supplementary Information:**

The online version contains supplementary material available at 10.1186/s12889-024-19089-2.

## Background

Menopausal women may experience discomfort owing to menopausal symptoms caused by changes in hormone levels. These symptoms affect various aspects of women's health, including physiological, psychological, and social aspects. From a physiological perspective, common symptoms include vasomotor symptoms [1–3], migraines [4], genitourinary symptoms with menopause [5], and osteoporosis [6]. From a psychological perspective, menopausal women may experience sleep problems [7, 8], depression [2], and anxiety [3]. At the social level, an existing study showed that approximately 40% of women experience menopausal symptoms-associated impaired work performance [9]. Menopausal women may consider seeking clinical assistance to alleviate the discomfort associated with this life stage.

Hormone therapy (HT) involves hormone supplementation to maintain the necessary hormonal balance in the body to alleviate the physiological discomfort caused by menopausal symptoms [10]. The HT regimen choice depends on hysterectomy history [11]. For patients with a history of hysterectomy, estrogen alone is recommended, whereas combined estrogen and progesterone is recommended for those without such a history [11]. In the 1990s, observational studies suggested the potential of HT in preventing chronic diseases such as cardiovascular diseases [12]. In response, the U.S. Food and Drug Administration mandated randomized controlled trials (RCTs) to confirm these potential cardiovascular benefits [12].

The Women's Health Initiative (WHI) HT trials, which commenced in 1993, comprised two parallel RCTs (one for women with an intact uterus and the other for women with hysterectomy) designed to evaluate the benefits and risks of HT in the prevention of chronic diseases among predominantly healthy postmenopausal women aged 50–79 years [13–15]. In 2002, after 5.2 years of follow-up, the estrogen plus progesterone trial was terminated early because of an increased risk of several diseases, including breast cancer, coronary heart disease, non-fatal stroke, venous thromboembolism, and overall cardiovascular disease [14]. HT-associated health risks outweigh the benefits of HT use in the prevention of fractures and colorectal cancer [14]. Therefore, there has been a substantial decline in the use of HT among women following the release of the 2002 WHI HT trial findings [16–23]. Moreover, Wu et al. documented a decrease in HT prescription rates among Taiwanese women aged 45–69, dropping from 21.6% in 2001 to 9.7% in 2004 [19].

While the estrogen plus progesterone trial was prematurely terminated, the postintervention follow-up for the two WHI HT trials continued. In 2017, Manson et al. presented the results of the 18-year follow-up study, revealing that HT use was not significantly associated with long-term risks of all-cause, cancer-related, or cardiovascular-related mortalities [24]. These findings contrast the perceived health disadvantages of HT stemming from the 2002 WHI HT trial findings, which garnered significant attention and affected the use of HT worldwide. Although descriptive studies conducted in the UK and Switzerland have shown an increasing trend in HT utilization after 2017 [25, 26], they did not quantify the effect of the 2017 WHI information on HT use.

However, to the best of our knowledge, there is no existing evidence regarding the information impact of the results of the 2017 postintervention follow-up on menopause-related visits and HT utilization. Therefore, this study aimed to evaluate the short-term effects of the 2017 WHI findings on outpatient visits and use of HT for menopause-related symptoms among women aged 50–60 years and HT in Taiwan. We employed an emulated cross-sectional study design to address methodological challenges of interval-censored data in this study.

## Methods

In the present study, we compared outpatient visits for menopause symptoms and HT use three months before and after the 2017 WHI study release. Conventional cross-sectional analysis cannot capture behavior changes. Ideally, a cohort study should be conducted to evaluate the 2017 WHI study's influence on HT discontinuation among women with menopause symptoms. Due to limitations in National Health Insurance claims data, we could not accurately track the onset and cessation of symptoms in individual women. This interval-censored data feature prevented us from identifying the menopause study population and distinguishing the reasons for stopping HT use. Consequently, conducting a cohort study to compare changes in HT use before and after the 2017 WHI study was not feasible. 

To overcome these challenges, we conducted an emulated repeated cross-sectional study, randomizing participants into either the exposure period (after the 2017 WHI study) or the non-exposure period (before the 2017 WHI study). As a result of this random assignment, interval-censored characteristics such as the onset and cessation of menopause were anticipated to be distributed similarly between both groups. Healthcare utilization was assessed during the study period. Additional information is provided in "Study design" section.

The study received approval from the Institutional Review Board (IRB) of National Yang Ming Chiao Tung University (IRB number: NYCU112074A). It adhered to the 1964 Declaration of Helsinki and its subsequent amendments, as well as the Strengthening the Reporting of Observational Studies in Epidemiology guidelines.

### Data source

Data was sourced from the National Health Insurance Research Database of the Health and Welfare Data Science Center, Ministry of Health and Welfare. Taiwan’s NHI is a form of social insurance that enrolls nearly 100% of the population. We used a dataset that included the Registry for Beneficiaries database, Ambulatory Care Expenditures by Visits database, Details of Ambulatory Care Orders database, Inpatient Expenditures by Admissions database, and Details of Inpatient Orders database. The Registry for Beneficiaries database contains personal insurance information including birth year, health insurance category, and insurance amounts. The other four datasets contain all medical claims of insured individuals, including disease diagnoses, procedures performed, and prescriptions dispensed during inpatient, outpatient, and emergency visits.

### Study design

**Fig. 1:**
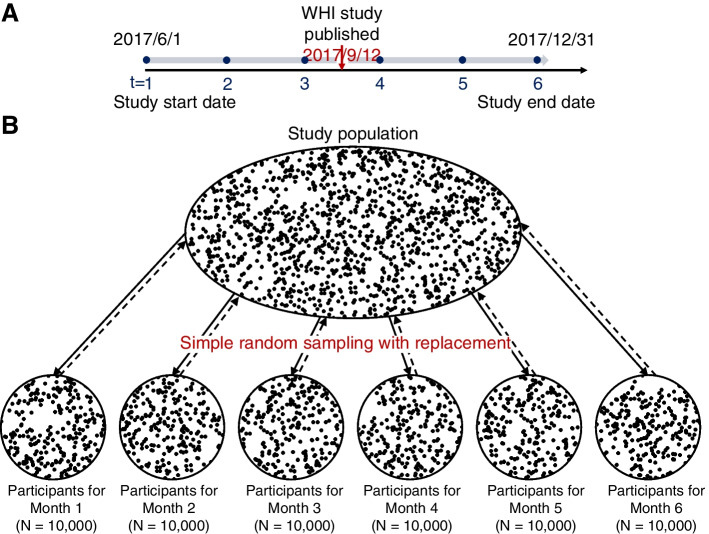
Study design and sampling approach. **A** Diagram illustrating the emulated cross-sectional study design. *WHI* Women’s Health Initiative. **B** Profile of sampling and randomization. We assume that the study population exists in each month of the study period

The study employed a 6-month repeated cross-sectional design, spanning June 2017 to December 2017. This timeframe included the 3 months before and 3 months after September 2017, denoted as the month of information exposure (Fig. 1A). From a female population aged 50–60 years (as described in "Study population" section), 10,000 women were randomly selected and assigned to a specific month. This procedure was repeated six times with replacement to establish six independent monthly groups (Fig. 1B) for emulating the randomization of information exposure.

### Study population

**Fig. 2:**
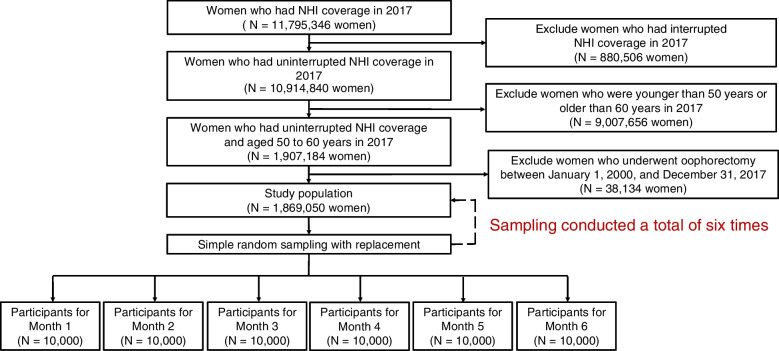
Flowchart for selection of the study participants. *NHI* National Health Insurance

Our target population comprised women aged 50–60 years with continuous NHI coverage between January and December 2017. We initially identified 10,914,840 women who had uninterrupted NHI coverage during the study period (Fig. 2). Of these, 1,907,084 were within 50–60 years of age. After excluding 38,134 women who underwent oophorectomy between January 1, 2000 and December 31, 2017, our final eligible study population comprised 1,869,050 women. We employed simple random sampling with replacement to select 10,000 study participants for each of the 6 months, resulting in a total of 60,000 participants for analysis.

### Exposure to the 2017 WHI study

The variable "exposure to the 2017 WHI study" was defined in relation to its publication month, specifically, September 2017, a pivotal period for information dissemination. The exposure status for each participant was determined based on their assignment month relative to September 2017, with study participants categorized as "yes" if assigned after that month and "no" if assigned before.

### Outcomes

In the present study, we assessed two primary outcome variables. The first one was outpatient visits related to menopausal symptoms; we investigated whether women aged 50–60 years had any outpatient visits for menopause-related symptoms during the designated month. The second was HT use; we examined whether women who had outpatient visits for menopausal symptoms were prescribed HT.

Regarding the first outcome, we identified an outpatient visit for menopause-related symptoms when women were diagnosed with primary diagnostic codes that included either the International Classification of Diseases, Ninth Revision, Clinical Modification (ICD-9-CM) code 627 or the International Classification of Diseases, Tenth Revision, Clinical Modification (ICD-10-CM) codes N92.4 and N95. Regarding the second outcome, we defined HT prescription as the receipt of any medications associated with the Anatomical Therapeutic Chemical codes G03C and G03F during the designated month.

### Covariates

Covariates potentially associated with HT use included demographic factors, medical history, healthcare provider attributes, and time trends. The demographic factors included age, income-related insurance premium amounts, health insurance category, and geographic location. The study participants were divided into two age groups: 50–54 and 55–60 years. NHI enrolment salary and health insurance category were determined based on the status in the assigned month. The income-related insurance premium amounts were divided into five categories: < NT$30,000, NT$30,001–NT$50,000, NT$50,001–NT$80,000, NT$80,001–NT$120,000, and ≥ NT$120,001 or above. Health insurance was classified into five categories following the National Health Insurance Act: Category 1: civil servants or employees of publicly or privately owned enterprises or institutions; Category 2: members of an occupational union who have no particular employers; Category 3: members of the Farmers’ Association or the Irrigation Association; Category 5: members of a household of low-income families; and Category 6: veterans and others (Category 4 is not available through the NHI database). Geographic location was determined based on the medical institutions most frequently visited by the study participants within 1 year before the first day of the randomly assigned month (index date) and was divided into six categories: north, central, south, east, outlying islands, and unknown.

Medical history included the following conditions: cardiovascular diseases (ICD-9-CM: 410–414, 430–437; ICD-10-CM: I20-I22, I24-I25, G45.0-G46.8, I60-I68), diabetes mellitus (ICD-9-CM: 250, 251.8, 357.2, 362.01, 362.02, 366.41, 583.81; ICD-10-CM: E08-E13), hyperlipidemia (ICD-9-CM: 272; ICD-10-CM: E71.30, E75.21-E75.22, E75.24, E75.3-E75.6, E77, E78.0-E78.6, E78.70, E78.79, E78.8-E78.9, E88), hypertension (ICD-9-CM: 401–405; ICD-10-CM: I10-I15, N26.2), liver diseases (ICD-9-CM: 570–573; ICD-10-CM: K70-K77), osteoporosis (ICD-9-CM: 733.01–733.09; ICD-10-CM: M81.0, M81.6, M81.8), breast cancer (ICD-9-CM: 174, 233.0, 238.3, 239.3; ICD-10-CM: C50, D05, D48.6, D49.3, Z51.12), and gynecological cancer (ICD-9-CM: 180, 182.0, 183.0; ICD-10-CM: C53, C54.1, C54.2, C54.3, C54.9, C56). Furthermore, medical history was defined as a history of more than two outpatient visits or more than one hospitalization record related to a specific condition in the 5 years preceding the index date.

Healthcare provider attributes included physician specialty, sex, and hospital ownership. These attributes were determined based on the characteristics of most outpatient visits for menopause-related symptoms during the assigned month. Time trend was defined as the natural progression of time during the study period, with each month serving as a unit of time.

### Statistical analysis

We employed descriptive statistics to describe the baseline characteristics of study participants (1) before and after the 2017 WHI study and (2) for each month. We evaluated the statistical significance of the differences between the study groups using the standardized mean difference (SMD), where SMD > 0.1 signified a significant difference.

We utilized logistic regression to investigate the impact of the 2017 WHI study on outpatient visits for menopause-related symptoms and HT use among women with outpatient visits. The variables of interest comprised a binary variable indicating exposure to the 2017 WHI report (${\text{X}}_{1}$) and its interaction with the time trend (${\text{X}}_{1}$ t). The binary variable "${\text{X}}_{1}$" signifies the immediate level change associated with the study publication; the interaction term "${\text{X}}_{1}$ t " represents the change over time following the study. In examining outpatient visits for menopause-related symptoms, we controlled for demographic factors, medical history, and time trend. In the analysis of HT use, the covariates included demographic factors, medical history, healthcare provider attributes, and time trend. SAS (version 9.4; SAS Institute, Inc., Cary, NC, USA) and R (version 4.2.2; R Foundation for Statistical Computing, Vienna, Austria) were used for data analysis.

### Sensitivity and scenario analyses

Sensitivity and scenario analyses were conducted to assess the robustness of the study design. The first sensitivity analysis involved random selection of a monthly sample of 30,000 participants over 6 months and logistic regression to assess the impact of the 2017 WHI study. In the second sensitivity analysis, bootstrapping was performed 100 times, following the same sampling method as in the first analysis, and 30,000 participants were randomly selected each month. This approach provided insights into the robustness of our effect estimates.

We performed a scenario analysis based on the 2002 WHI HT trial findings, which closely mirrored our base case study in design and statistical analysis. The study period spanned from January 2002 to January 2003, excluding the exposure month (July 2002), covering the 6 months before and after the exposure. This scenario serves as a positive control to validate our study design.

## RESULTS

### Demographic characteristics

The baseline characteristics of the study participants (*N* = 60,000 women) before and after the 2017 WHI study are summarized in Table 1 and those for each month are presented in Additional File 1. The average age of the study participants was approximately 55 years. Over 80% of the study participants reported an NHI enrolment salary below NT$50,000, and nearly half of them belonged to the first category of health insurance, which includes those employed by the government, schools, or privately operated public utility enterprises. Most study participants lived in the northern region, followed by the southern and central regions. Regarding medical history, < 25% of the study participants had an existing condition, such as hyperlipidemia, hypertension, diabetes mellitus, liver disease, cardiovascular disease, and breast or gynecological cancer. The observed baseline characteristics among the included participants were similar and comparable (SMD < 0.1) before and after the 2017 WHI study, as well as for each month.

**Table 1:** Baseline characteristics of study participants before and after publication of the 2017 WHI Study

	Study participants before publication of the WHI study in 2017 (*N *= 30,000)		Study participants after publication of the WHI study in 2017 (*N* = 30,000)		SMD	*p*-value ^*b*^
	*N*	%	*N*	%		
**Demographic factors**						
					0.01	0.13
Age (mean ± S.D., years)	54.92 ± 3.11		54.92 ± 3.11			
50-54	16,803	56.01	16,654	55.51	0.01	0.22
55-60	13,197	43.99	13,346	44.49		
Income-related insurance premium amounts (NT$)					<0.01	0.98
≤30,000	16,781	55.94	16,729	55.76		
30,001-50,000	9,778	32.59	9,817	32.72		
50,001-80,000	2,161	7.20	2,183	7.28		
80,001-120,000	952	3.17	936	3.12		
≥120,001	328	1.09	335	1.12		
Categories of health Insurance ^*a*^					<0.01	0.27
Category 1	13,942	46.47	14,158	47.19		
Category 2	8,883	29.61	8,686	28.95		
Category 3	3,052	10.17	3,041	10.14		
Category 5	172	0.57	193	0.64		
Category 6	3,951	13.17	3,922	13.07		
Geographic area					<0.01	0.2
Northern	13,081	43.60	13,157	43.86		
Central	6,568	21.89	6,386	21.29		
Southern	7,457	24.86	7,466	24.89		
Eastern	555	1.85	611	2.04		
Outlying islands	164	0.55	146	0.49		
Unknown	2,175	7.25	2,234	7.45		
**Medical history**						
Cardiovascular disease	2,488	8.29	2,634	8.78	0.02	0.03
Diabetes mellitus	3,583	11.94	3,665	12.22	<0.01	0.3
Hyperlipidemia	7,247	24.16	7,441	24.80	0.02	0.07
Hypertension	7,044	23.48	7,277	24.26	0.02	0.03
Liver disease	3,128	10.43	3,125	10.42	<0.01	0.97
Osteoporosis	726	2.42	721	2.40	<0.01	0.89
Breast cancer	733	2.44	749	2.50	<0.01	0.67
Gynecological cancer	233	0.78	273	0.91	0.02	0.07

^*a*^The Registry for Beneficiaries Database cannot be accessed for the fourth insurance category

^*b*^For statistical tests, we conducted t-tests for continuous variables and chi-square tests for categorical variables

### Base case analysis

Tables 2 and 3 show the results of logistic regressions assessing the impact of the 2017 WHI study on outpatient visits for menopause-related symptoms and HT use among women who had outpatient visits, respectively. Crude and adjusted models consistently demonstrated that the 2017 report did not have a significant impact on outpatient visits for menopause-related symptoms (adjusted odds ratio [aOR] of level change: 1.20, 95% CI: 0.85–1.71; aOR of slope change: 1.11, 95% CI: 0.92–1.35; Table 2) or HT use among women who had outpatient visits (aOR of level change: 1.08, 95% CI: 0.49–2.4; aOR of slope change: 1.37, 95% CI: 0.88–2.12; Table 3).

**Table 2:** Logistic regression analysis of the impact of 2017 WHI study on outpatient visits for menopause-related symptoms among women aged 50-60 years

	Crude model	Adjusted model ^*a*^
	OR (95% CI)	OR (95% CI)
2017 WHI study (X_1_)	1.20 (0.85-1.70)	1.20 (0.85-1.71)
2017 WHI study × Time trend ( X_1_t)	1.12 (0.92-1.35)	1.11 (0.92-1.35)
Time trend (t)	0.91 (0.80-1.05)	0.91 (0.80-1.05)
**Demographic factors**		
Age (years)		0.96 (0.94-0.99)
Income-related insurance premium amounts (NT$)		
≤30,000		ref.
30,001-50,000		1.16 (0.95-1.40)
50,001-80,000		0.80 (0.55-1.16)
80,001-120,000		1.04 (0.65-1.68)
≥120,001		1.20 (0.59-2.47)
Categories of health insurance ^*b*^		
Category 1		ref.
Category 2		0.97 (0.80-1.18)
Category 3		1.03 (0.76-1.39)
Category 5		1.28 (0.52-3.15)
Category 6		1.10 (0.83-1.46)
Geographic area		
Northern		ref.
Central		1.18 (0.97-1.44)
Southern		1.10 (0.90-1.33)
Eastern		1.12 (0.65-1.93)
Outlying islands		1.21 (0.45-3.27)
Unknown		0.09 (0.03-0.23)
**Medical History**		
Cardiovascular disease		1.36 (1.05-1.76)
Diabetes mellitus		0.85 (0.65-1.11)
Hyperlipidemia		1.24 (1.02-1.52)
Hypertension		0.76 (0.62-0.93)
Liver disease		1.22 (0.96-1.55)
Osteoporosis		2.33 (1.65-3.29)
Breast cancer		0.23 (0.09-0.63)
Gynecological cancer		0.35 (0.09-1.41)

*95%* *CI* 95% confidence interval, *NT$* New Taiwan Dollar, *OR* odds ratio, *ref* reference, *WHI *Women’s Health Initiative

^*a*^The adjusted model has been controlled for time trend, age, income-related insurance premium amounts, health insurance categories, geographic area, cardiovascular disease, diabetes mellitus, hyperlipidemia, hypertension, liver disease, osteoporosis, breast cancer, and gynecological cancer

^*b*^The Registry for Beneficiaries Database cannot be accessed for the fourth insurance category

**Table 3:** Logistic regression analysis of the impact of 2017 WHI study on HT use among women who had outpatient visits

	Crude model	Adjusted model ^*a*^
	OR (95% CI)	OR (95% CI)
2017 WHI study (X_1_)	1.02 (0.48-2.21)	1.08 (0.49-2.40)
2017 WHI study × Time trend (X_1_t)	1.31 (0.86-2.01)	1.37 (0.88-2.12)
Time trend (t)	0.88 (0.65-1.20)	0.85 (0.62-1.18)
**Demographic factors**		
Age (years)		1.01 (0.95-1.07)
Income-related insurance premium amounts (NT$)		
≤30,000		ref.
30,001-50,000		0.75 (0.48-1.16)
≥50,001		0.50 (0.26-0.96)
Categories of health insurance ^*b*^		
Category 1		ref.
Category 2		1.12 (0.72-1.74)
Category 3		1.00 (0.50-2.00)
Category 5		1.93 (0.20-18.59)
Category 6		0.88 (0.47-1.65)
Geographic area		
Northern		ref.
Central		1.29 (0.82-2.04)
Southern		1.35 (0.87-2.09)
Eastern, outlying islands, or unknown		1.15 (0.43-3.11)
**Medical history**		
Cardiovascular disease		1.46 (0.79-2.67)
Diabetes mellitus		0.67 (0.40-1.11)
Hyperlipidemia		0.84 (0.47-1.53)
Hypertension		0.69 (0.45-1.06)
Liver disease		0.89 (0.57-1.38)
**Healthcare Provider Attributes**		
Physician specialty		
Obstetrician or gynecologist		ref.
Other specialty		1.15 (0.62-2.11)
Physician gender		
Male physician		ref.
Female physician or unknown		0.91 (0.60-1.39)

*95%* *CI* 95% confidence interval, *NT$* New Taiwan Dollar, *OR *odds ration,* ref* reference, *WHI* Women's Health Initiative

^*a*^The adjusted model has been controlled for time trend, age, income-related insurance premium amounts, health insurance categories, geographic area, cardiovascular disease, diabetes mellitus, hyperlipidemia, hypertension, liver disease, physician specialty, and physician sex

^*b*^The Registry for Beneficiaries cannot be accessed for the fourth insurance category

### Base case sensitivity analysis

The two sensitivity analyses involving (1) sampling of 30,000 participants each month and (2) sampling of 30,000 participants each month with bootstrapping for 100 iterations consistently indicated that the 2017 WHI study did not have a significant effect on outpatient visits for menopause-related symptoms (sensitivity analysis (1): aOR of level change: 0.95, 95% CI: 0.78–1.15; aOR of slope change: 1.03, 95% CI: 0.95–1.11; sensitivity analysis (2): aOR of level change: 0.99, 95% CI: 0.77–1.21; aOR of slope change: 0.96, 95% CI: 0.87–1.06) or HT use among participants who had outpatient visits (sensitivity analysis (1): aOR of level change: 0.92, 95% CI: 0.59–1.43; aOR of slope change: 1.07, 95% CI: 0.83–1.38; sensitivity analysis (2): aOR of level change: 1.03, 95% CI: 0.67–1.55; aOR of slope change: 0.99, 95% CI: 0.8–1.26; Table 4). These results are consistent with those of the base case analysis.

**Table 4:** Sensitivity analysis of the impact of 2017 WHI study on outpatient visits and HT use

	Base case analysis: Random sampling of 10,000 participants each month	Sensitivity analysis (1): Random sampling of 30,000 participants each month	Sensitivity analysis (2): Random sampling of 30,000 participants each month, with 100 bootstrap iterations
	aOR (95% CI)	aOR (95% CI)	aOR^a^ (95% CI)
Outcome 1: The outpatient visits for menopause-related symptoms among women aged 50 to 60^b^			
2017 WHI study (${\text{X}}_{1}$)	1.20 (0.85–1.71)	0.95 (0.78–1.15)	0.99 (0.77–1.21)
2017 WHI study × Time trend (${\text{X}}_{1}$t)			
	1.11 (0.92–1.35)	1.03 (0.95–1.11)	0.96 (0.87–1.06)
Time trend (t)	0.91 (0.80–1.05)	0.92 (0.82–1.03)	1.01 (0.93–1.09)
Outcome 2: The use of HT among women aged 50 to 60 who had outpatient visits^c^			
2017 WHI study (${\text{X}}_{1}$)	1.08 (0.49–2.40)	0.92 (0.59–1.43)	1.03 (0.67–1.55)
2017 WHI study × Time trend (${\text{X}}_{1}$t)			
	1.37 (0.88–2.12)	1.07 (0.83–1.38)	0.99 (0.80–1.26)
Time trend (t)	0.85 (0.62–1.18)	1.05 (0.88–1.25)	1.01 (0.85–1.20)

*aOR* adjusted odds ratio, *95% CI* 95% confidence interval, *HT* Hormone therapy, *WHI* Women’s Health Initiative

^*a*^ Median aOR obtained from bootstrapping 100 iterations

^*b*^ The outcome 1 adjusted model has been controlled for time trend, age, income-related insurance premium amounts, categories of health insurance, geographic area, cardiovascular disease, diabetes mellitus, hyperlipidemia, hypertension, liver disease, osteoporosis, breast cancer, and gynecological cancer

^*c*^The outcome 2 adjusted model has been controlled for time trend, age, income-related insurance premium amounts, categories of health insurance, geographic area, cardiovascular disease, diabetes mellitus, hyperlipidemia, hypertension, liver disease, physician specialty, and physician sex

### Scenario analysis

In our scenario analysis, we adopted an identical study design to validate our findings by assessing the effects of the 2002 WHI HT trial. The 2002 report was associated with reduced likelihood of outpatient visits for menopause-related symptoms among women aged 50–60 years, with an aOR of 0.67 (95% CI: 0.62–0.73) and a negative change over time (aOR: 0.95, 95% CI: 0.93–0.98; Additional File 2). However, the 2002 report was associated with increased odds of HT use among women who had outpatient visits, with an aOR of 2.08 (95% CI: 1.6–2.69) and a positive change over time (aOR: 1.24, 95% CI: 1.15–1.35; Additional File 2).

## Discussion

### Summary of key findings

We introduced an emulated cross-sectional study design to investigate the impact of the 2017 WHI study on HT use in Taiwan. Our findings show that the 2017 WHI study had no significant impact on the level or slope of menopause-related outpatient visits among middle-aged women and their use of HT. The sensitivity analysis further underscored the robustness of these estimates. There are two possible explanations for the lack of a significant effect of the 2017 WHI study on outpatient visits or HT use among women who had outpatient visits. First, although the updated information released in the 2017 WHI study reduced concerns about the overall mortality associated with HT, its impact remained limited and did not fully counteract negative health risks, such as cancer incidence, as highlighted in the 2002 WHI study [27, 28]. Second, there was a temporal gap between the publication of the study results and the update of Taiwan’s menopausal treatment guidelines. The Taiwanese Menopause Society released updated guidelines in 2019, incorporating the 2017 WHI study findings into their recommendations [10]. Although this time lag may not affect the treatment choice of healthcare providers, it may reduce the demand for HT use among women experiencing menopausal symptoms, particularly those with mild symptoms.

Our scenario analysis revealed a significant negative effect of the 2002 WHI HT trial findings on overall HT use among women aged 50–60 years, consistent with the results of previous studies ([16–20] (Additional File 3). This reduction in HT use can be primarily attributed to a decrease in menopause-related outpatient visits. Notably, the 2002 WHI HT trial had a negative effect on outpatient visits among women aged 50–60 years but a positive effect on HT use among women who had outpatient visits (Additional File 2). One possible explanation is that the risks associated with HT have long been discussed by various women's health associations and have received extensive media coverage in Taiwan before the 2002 WHI publication [29]. Consequently, women may have been more inclined not to visit physicians [29]. Those who sought menopause-related outpatient care may have experienced more severe menopausal symptoms, thereby exhibiting a greater tendency to opt for HT for symptom management.

### Limitations

This study had two limitations, which are related to the database and research design. Firstly, because of the inherent constraints of the NHI claims database in identifying women with menopausal symptoms, we used women aged 50–60 years as proxies for those with menopausal symptoms. Consequently, women without menopausal symptoms may have been included in this study. Secondly, we excluded women who had a history of oophorectomy between January 1, 2000, and December 31, 2017. This exclusion criterion may have resulted in cases where individuals had not yet undergone oophorectomy at the time of assignment for a given month, leading to their unintended exclusion from the observation.

Although these limitations may influence sample selection, the approach of random sampling and assignment employed in this study may ensure that a potential selection bias is randomly distributed across the study periods, including 3 months before and after the publication of the 2017 WHI study. Furthermore, the sensitivity analysis results derived from 100 bootstrapped iterations were consistent with the base case analysis results, emphasizing the robustness of the research findings. Therefore, these measurement errors are unlikely to introduce a significant bias in estimating the impact of the 2017 WHI study findings.

### Strengths

This study introduced an innovative approach to emulate a cross-sectional study for simulating the randomization of information shock, aimed at addressing two methodological challenges. The first challenge arose from the limitation of the NHI database in identifying the study population, resulting from variations in menopausal symptoms among women and unavailability of records in claims data in cases where women do not seek medical attention for these symptoms. The second challenge relates to the natural development of menopause, which evolves over time. If we had employed a conventional cohort, monitoring study participants before and after the publication of the 2017 WHI study, it would have been difficult to distinguish whether the observed changes were due to the natural development of menopause or the impact of the 2017 WHI study findings. These issues may have affected the internal validity of the study.

To address these challenges, we adopted an emulated repeated cross-sectional design with random sampling and assignments to emulate the randomization of the 2017 information shock. This research design randomized women aged 50–60 years and accounted for unobserved confounders before and after the publication of the 2017 WHI study findings. Additionally, descriptive statistics highlighted that the distribution of baseline characteristics among the study participants, generated through random sampling and assignments, was similar. The inferential statistics indicated minimal differences between the crude and adjusted models. These results confirmed the robustness of the study design.

### Recommendations for future studies

Because of constraints related to the accessibility of NHI claims data during the study, the primary emphasis was evaluation of the short-term impact of the 2017 WHI study. Considering Rogers' diffusion of innovation theory, which suggests that the dissemination of innovative information takes time, it is possible that a time lag effect exists [30]. To gain a more comprehensive understanding, future research should aim to investigate the long-term effects of the 2017 WHI study findings.

In addition, it is noteworthy that a temporal gap existed between the publication of the 2017 WHI study and the release of updated treatment guidelines in Taiwan. Therefore, subsequent studies can investigate whether a significant shift occurred in 2019 in response to the updated guidelines.

Furthermore, for future observational follow-up studies, it may be beneficial to consider the approach employed in this study, which involved emulating cross-sectional data to simulate a randomization of information shock. This methodology proves particularly useful when dealing with interval-censored data and addressing the challenges of distinguishing the effect of an intervention effect from the natural progression of a disease.

## Conclusions

The 2017 WHI study had no demonstrable effect on the level and rate of change in outpatient visits among middle-aged menopausal women and HT use within this group. This implies that the updated findings of the 2017 WHI HT study may not have fully counteracted the enduring negative effect of the 2002 WHI HT trial on HT use. In future observational studies involving interval-censored data, researchers may consider employing the emulated randomization approach used in this study to establish a repeated cross-sectional design for before-and-after comparisons.

### Supplementary Information

Additional File 1: Baseline characteristics of the study participants for each month (*N* = 60,000).

Additional File 2: Scenario analysis of the impact of the 2002 WHI study on outpatient visits for menopause-related symptoms and HT use among women aged 50–60 years.

Additional File 3: Scenario analysis of the impact of the 2002 WHI study on the overall use of HT among women aged 50–60 years (*N* = 120,000).

## Data Availability

The data supporting the findings of this study are available from the Ministry of Health and Welfare, Taiwan; however, restrictions apply to the availability of these data, which were used under the license for the current study and are not publicly available. The code supporting the findings of this study is available from the corresponding authors upon request.

## Abbreviations

HT: Hormone therapy
WHI: Women’s Health Initiative
RCT: Randomized controlled trial
NHI: National Health Insurance
IRB: Institutional Review Board
ICD-9-CM: International Classification of Diseases, Ninth Revision, Clinical Modification
ICD-10-CM: International Classification of Diseases, Tenth Revision, Clinical Modification
SMD: Standardized mean difference
aOR: Adjusted odds ratio
