# Supplementary material for: The 2017 Women’s Health Initiative study and use of hormone therapy: an emulated repeated cross-sectional study
Source: BMC Public Health. 2024 Jun 24;24:1674. doi: 10.1186/s12889-024-19089-2 (PMC11194959; doi:10.1186/s12889-024-19089-2)
Supplement: Supplementary file 1 — Additional File 1: Baseline characteristics of the study participants for each month (N = 60,000). [file 12889_2024_19089_MOESM1_ESM.docx]

**ADDITIONAL FILES**

**The** **2017 Women’s Health Initiative study and use of hormone therapy: an emulated repeated cross-sectional study**

Chen-Han Chueh, Pei-Kuan Ho, Wai-Hou Li, Ming-Neng Shiu, I-Ting Wang, Yu-Wen Wen, Yi-Wen Tsai

*Correspondence:

Yi-Wen Tsai

National Yang Ming Chiao Tung University

No. 155, Section 2, Linong St., Beitou District

Taipei, Taiwan 112304
[ywtsai@nycu.edu.tw](mailto:ywtsai@nycu.edu.tw)

Chen-Han Chueh

National Yang Ming Chiao Tung University

No. 155, Sec. 2, Linong St., Beitou District

Taipei, Taiwan 112304
[chchueh.y@nycu.edu.tw](mailto:chchueh.y@nycu.edu.tw)

**Additional File 1**: Baseline characteristics of the study participants for each month (N = 60,000)

|  | Participants  for month 1  (N = 10,000) | | Participants  for month 2  (N = 10,000) | | Participants  for month 3  (N = 10,000) | | Participants  for month 4  (N = 10,000) | | Participants  for month 5  (N = 10,000) | | Participants  for month 6  (N = 10,000) | | SMD | *p*-value |  |
| --- | --- | --- | --- | --- | --- | --- | --- | --- | --- | --- | --- | --- | --- | --- | --- |
|  |  |  |  |  |  |  |  |  |  |  |  |  |  |  |  |
|  | N | % | N | % | N | % | N | % | N | % | N | % |  |  |  |
| **Demographic factors** |  |  |  |  |  |  |  |  |  |  |  |  |  | |  |
| Age (mean ± S.D., year) | 54.90 ± 3.12 | | 54.95 ± 3.10 | | 54.90 ± 3.10 | | 54.93 ± 3.09 | | 55.02 ± 3.10 | | 54.91 ± 3.13 | | 0.02 | 0.04 |  |
| Age |  |  |  |  |  |  |  |  |  |  |  |  | 0.01 | 0.44 |  |
| 50-54 | 5,612 | 56.12 | 5,587 | 55.87 | 5,604 | 56.04 | 5,576 | 55.76 | 5,480 | 54.80 | 5,598 | 55.98 |  |  |  |
| 55-60 | 4,388 | 43.88 | 4,413 | 44.13 | 4,396 | 43.96 | 4,424 | 44.24 | 4,520 | 45.20 | 4,402 | 44.02 |  | |  |
| Income-related insurance premium amounts (NT$) | | |  |  |  |  |  |  |  |  |  |  | <0.01 | 0.91 |  |
| ≤30,000 | 5,604 | 56.04 | 5,619 | 56.19 | 5,558 | 55.58 | 5,580 | 55.80 | 5,566 | 55.66 | 5,583 | 55.83 |  |  |  |
| 30,001-50,000 | 3,230 | 32.30 | 3,238 | 32.38 | 3,310 | 33.10 | 3,264 | 32.64 | 3,315 | 33.15 | 3,238 | 32.38 |  | |  |
| 50,001-80,000 | 732 | 7.32 | 689 | 6.89 | 740 | 7.40 | 738 | 7.38 | 703 | 7.03 | 742 | 7.42 |  | |  |
| 80,001-120,000 | 323 | 3.23 | 340 | 3.40 | 289 | 2.89 | 311 | 3.11 | 304 | 3.04 | 321 | 3.21 |  | |  |
| ≥120,001 | 111 | 1.11 | 114 | 1.14 | 103 | 1.03 | 107 | 1.07 | 112 | 1.12 | 116 | 1.16 |  | |  |
| Categories of health insurance ^a^ |  |  |  |  |  |  |  |  |  |  |  |  | <0.01 | 0.68 |  |
| Category 1 | 4,642 | 46.42 | 4,651 | 46.51 | 4,649 | 46.49 | 4,743 | 47.43 | 4,663 | 46.63 | 4,752 | 47.52 |  |  |  |
| Category 2 | 2,995 | 29.95 | 2,945 | 29.45 | 2,943 | 29.43 | 2,874 | 28.74 | 2,899 | 28.99 | 2,913 | 29.13 |  | |  |
| Category 3 | 997 | 9.97 | 1,039 | 10.39 | 1,016 | 10.16 | 1,021 | 10.21 | 1,049 | 10.49 | 971 | 9.71 |  | |  |
| Category 5 | 55 | 0.55 | 68 | 0.68 | 49 | 0.49 | 70 | 0.70 | 59 | 0.59 | 64 | 0.64 |  | |  |
| Category 6 | 1,311 | 13.11 | 1,297 | 12.97 | 1,343 | 13.43 | 1,292 | 12.92 | 1,330 | 13.30 | 1,300 | 13.00 |  | |  |
| Geographic area |  |  |  |  |  |  |  |  |  |  |  |  | 0.02 | 0.19 |  |
| Northern | 4,353 | 43.53 | 4,449 | 44.49 | 4,279 | 42.79 | 4,357 | 43.57 | 4,422 | 44.22 | 4,378 | 43.78 |  |  |  |
| Central | 2,215 | 22.15 | 2,140 | 21.40 | 2,213 | 22.13 | 2,163 | 21.63 | 2,102 | 21.02 | 2,121 | 21.21 |  | |  |
| Southern | 2,468 | 24.68 | 2,444 | 24.44 | 2,545 | 25.45 | 2,440 | 24.40 | 2,522 | 25.22 | 2,504 | 25.04 |  | |  |
| Eastern | 190 | 1.90 | 204 | 2.04 | 161 | 1.61 | 208 | 2.08 | 204 | 2.04 | 199 | 1.99 |  | |  |
| Outlying islands | 47 | 0.47 | 59 | 0.59 | 58 | 0.58 | 45 | 0.45 | 49 | 0.49 | 52 | 0.52 |  | |  |
| Unknown | 727 | 7.27 | 704 | 7.04 | 744 | 7.44 | 787 | 7.87 | 701 | 7.01 | 746 | 7.46 |  | |  |
| **Medical history** |  |  |  |  |  |  |  |  |  |  |  |  |  | |  |
| Cardiovascular disease | 812 | 8.12 | 862 | 8.62 | 814 | 8.14 | 864 | 8.64 | 891 | 8.91 | 879 | 8.79 | 0.01 | 0.22 |  |
| Diabetes mellitus | 1,201 | 12.01 | 1,234 | 12.34 | 1,148 | 11.48 | 1,214 | 12.14 | 1,262 | 12.62 | 1,189 | 11.89 | 0.02 | 0.21 |  |
| Hyperlipidemia | 2,396 | 23.96 | 2,499 | 24.99 | 2,352 | 23.52 | 2,458 | 24.58 | 2,537 | 25.37 | 2,446 | 24.46 | 0.02 | 0.03 |  |
| Hypertension | 2,333 | 23.33 | 2,368 | 23.68 | 2,343 | 23.43 | 2,370 | 23.70 | 2,489 | 24.89 | 2,418 | 24.18 | 0.02 | 0.1 |  |
| Liver disease | 1,085 | 10.85 | 1,052 | 10.52 | 991 | 9.91 | 1,047 | 10.47 | 1,051 | 10.51 | 1,027 | 10.27 | 0.01 | 0.39 |  |
| Osteoporosis | 248 | 2.48 | 231 | 2.31 | 247 | 2.47 | 239 | 2.39 | 244 | 2.44 | 238 | 2.38 | <0.01 | 0.97 |  |
| Breast cancer | 234 | 2.34 | 236 | 2.36 | 263 | 2.63 | 249 | 2.49 | 253 | 2.53 | 247 | 2.47 | <0.01 | 0.79 |  |
| Gynecological cancer | 70 | 0.70 | 93 | 0.93 | 70 | 0.70 | 85 | 0.85 | 95 | 0.95 | 93 | 0.93 | 0.02 | 0.15 |  |

*NT$* New Taiwan Dollar, *SMD* standardized mean difference

^a^ The Registry for Beneficiaries Database cannot be obtained for the fourth insurance category.
