# Supplementary material for: The 2017 Women’s Health Initiative study and use of hormone therapy: an emulated repeated cross-sectional study
Source: BMC Public Health. 2024 Jun 24;24:1674. doi: 10.1186/s12889-024-19089-2 (PMC11194959; doi:10.1186/s12889-024-19089-2)
Supplement: Supplementary file 2 — Additional File 2: Scenario analysis of the impact of the 2002 WHI study on outpatient visits for menopause-related symptoms and HT use among women aged 50–60 years. [file 12889_2024_19089_MOESM2_ESM.docx]

**ADDITIONAL FILES**

**The** **2017 Women’s Health Initiative study and use of hormone therapy: an emulated repeated cross-sectional study**

Chen-Han Chueh, Pei-Kuan Ho, Wai-Hou Li, Ming-Neng Shiu, I-Ting Wang, Yu-Wen Wen, Yi-Wen Tsai

*Correspondence:

Yi-Wen Tsai

National Yang Ming Chiao Tung University

No. 155, Section 2, Linong St., Beitou District

Taipei, Taiwan 112304
[ywtsai@nycu.edu.tw](mailto:ywtsai@nycu.edu.tw)

Chen-Han Chueh

National Yang Ming Chiao Tung University

No. 155, Sec. 2, Linong St., Beitou District

Taipei, Taiwan 112304
[chchueh.y@nycu.edu.tw](mailto:chchueh.y@nycu.edu.tw)

**Additional File 2**: Scenario analysis of the impact of the 2002 WHI study on outpatient visits for menopause-related symptoms and HT use among women aged 50–60 years

|  | Crude model | Adjusted model |
| --- | --- | --- |
|  | OR (95% CI) | OR (95% CI) |
| Outcome 1: The outpatient visits for menopause-related symptoms among women aged 50 to 60 years ^a^  (N = 120,000) | | |
| 2002 WHI study ($X_{1}$) | 0.69 (0.64-0.76)  0.95 (0.93-0.98) | 0.67 (0.62-0.73)  0.95 (0.93-0.98) |
| 2002 WHI study × Time trend ($X_{1}$t) |  |  |
| Time trend (t) | 1.02 (0.999-1.03) | 1.02 (1.004-1.04) |
| Outcome 2: The usage of HT among women aged 50 to 60 who had outpatient visits ^b^ (N = 9,071) | | |
| 2002 WHI study ($X_{1}$) | 2.00 (1.55-2.57)  1.25 (1.15-1.35) | 2.08 (1.60-2.69)  1.24 (1.15-1.35) |
| 2002 WHI study × Time trend ($X_{1}$t) |  |  |
| Time trend (t) | 0.82 (0.78-0.87) | 0.82 (0.78-0.86) |

*95% CI* 95% confidence interval, *HT* hormone therapy, *OR* odds ratio, *WHI* Women’s Health Initiative

^a^ The outcome 1 adjusted model has been controlled for time trend, age, income-related insurance premium amounts, categories of health insurance, geographic area, cardiovascular disease, diabetes mellitus, hyperlipidemia, hypertension, liver disease, osteoporosis, breast cancer, and gynecological cancer.

^b^ The outcome 2 adjusted model has been controlled for time trend, age, income-related insurance premium amounts, categories of health insurance, geographic area, cardiovascular disease, diabetes mellitus, hyperlipidemia, hypertension, liver disease, osteoporosis, physician specialty, physician sex, and hospital ownership.
