# Supplementary material for: The 2017 Women’s Health Initiative study and use of hormone therapy: an emulated repeated cross-sectional study
Source: BMC Public Health. 2024 Jun 24;24:1674. doi: 10.1186/s12889-024-19089-2 (PMC11194959; doi:10.1186/s12889-024-19089-2)
Supplement: Supplementary file 3 — Additional File 3: Scenario analysis of the impact of the 2002 WHI study on the overall use of HT among women aged 50–60 years (N = 120,000). [file 12889_2024_19089_MOESM3_ESM.docx]

**ADDITIONAL FILES**

**The** **2017 Women’s Health Initiative study and use of hormone therapy: an emulated repeated cross-sectional study**

Chen-Han Chueh, Pei-Kuan Ho, Wai-Hou Li, Ming-Neng Shiu, I-Ting Wang, Yu-Wen Wen, Yi-Wen Tsai

*Correspondence:

Yi-Wen Tsai

National Yang Ming Chiao Tung University

No. 155, Section 2, Linong St., Beitou District

Taipei, Taiwan 112304
[ywtsai@nycu.edu.tw](mailto:ywtsai@nycu.edu.tw)

Chen-Han Chueh

National Yang Ming Chiao Tung University

No. 155, Sec. 2, Linong St., Beitou District

Taipei, Taiwan 112304
[chchueh.y@nycu.edu.tw](mailto:chchueh.y@nycu.edu.tw)

**Additional File 3**: Scenario analysis of the impact of the 2002 WHI study on the overall use of HT among women aged 50–60 years (N = 120,000)

|  | Crude model | Adjusted model ^a^ |
| --- | --- | --- |
|  | OR (95% CI) | OR (95% CI) |
| 2002 WHI study ($X_{1}$) | 0.76 (0.69-0.83) | 0.74 (0.68-0.81) |
| 2002 WHI study × Time trend ($X_{1}$t) | 0.98 (0.95-1.002) | 0.98 (0.95-1.01) |
| Time trend (t) | 0.99 (0.98-1.01) | 1.00 (0.98-1.02) |

*95% CI* 95% confidence interval, *HT* hormone therapy, *OR* odds ratio, *WHI* Women’s Health Initiative

^a^ The adjusted model has been controlled for time trends, age, income-related insurance premium amounts, categories of health insurance, geographic area, breast cancer, cardiovascular disease, venous thromboembolism, liver disease, diabetes mellitus, hyperlipidemia, hypertension, osteoporosis, and gynecological cancer.
